# Mental health, suicide attempt, and family function for adolescents’ primary health care during the COVID-19 pandemic

**DOI:** 10.12688/f1000research.109603.1

**Published:** 2022-05-16

**Authors:** Indiana-Luz Rojas-Torres, Mostapha Ahmad, Juan Manuel Martín Álvarez, Antonio A Golpe, Richard de Jesús Gil Herrera

**Affiliations:** 1 Universidad Simón Bolívar, Facultad Ciencias de la Salud, Barranquilla, Colombia; 2 Universidad Americana de Europa (UNADE), Cancún, Mexico; 3 International University of La Rioja, Logroño, Spain; 4 University of Huelva, Huelva, Spain

**Keywords:** Adolescent, Mental Health, Family Relationships, attempted suicide, Primary care, clustering algorithms

## Abstract

**Background: **The study’s purpose was to identify associations between mental health risk, suicide attempts, and family function.

**Methods:** A correlational, descriptive, and cross-sectional study was carried out in a group of adolescents in the last grade of secondary school to establish the association between mental health risk, suicide attempt, and family functionality. The instruments used were the self-report questionnaire, the suicide risk assessment scale, and the family APGAR. Data analysis was performed using the artificial intelligence algorithm (gower clustering).

**Results: **246 adolescents responded to the three instruments, which made it possible to select those with correlations of sensitive interest and, based on these, an intervention plan. Psychological distress was found in 28%, psychotic symptoms in 85%, and problematic alcohol use in 9%. Good family functioning was identified in 34% and some type of family dysfunction in 66%. In terms of suicide risk, there was a low suicide risk of 74%, 24% medium risk, and 2% high risk. It could be shown that there is a correlation in a group of 15% of the respondents.

**Conclusions: **The risk of suffering mental health deterioration and the suicide risk, during this pandemic period, seems to be related to family functionality.

## Introduction

The World Health Organization (WHO) conceives mental health as the state of well-being of a person for the performance of their activities of daily life in the environment in which they are, overcoming the stressors that can be seen exposed ( WHO, 2001). Good mental health allows the individual to maintain balance and harmony both individually and as a group ( Loaiza *et al.,* 2019).

During the life course of early and intermediate adolescence, which includes the ages between 12 to 18 years ( Ministry of Health, 2016), and late adolescence, which can extend to 21 years ( Brittany Allen, 2012), changes occur of a biological, psychological, and socio-cultural nature ( Garcia *et al.,* 2015), in which manifestations related to depression, anxiety, eating disorders, and negative feelings that can lead to harmful behaviors such as self-destructive thoughts and suicidal ideation that are frequent ( Texeira *et al.*, 2020), and those are considered as the main risks of suicidal behavior itself ( Canón and Carmona 2018).

According to the WHO (2016), depression is the most common mental disorder, affecting more than 350 million people worldwide. Mental disorders, state the same organization, represent 16% of the global burden of diseases in people aged 10 to 19, with suicide being the third leading cause of death in young people aged 15 to 19 ( WHO, 2019).

In the case of Colombia, the number of people between the ages of 0 and 19 years who consult for mental disorders is increasing every day. According to data from the 2003 National Mental Health Survey, during adolescence there is an increase in the frequency of symptoms related to mental health, the most common being anxiety, phobia, anguish, post-traumatic stress, and panic disorder ( Ministry of Health, 2003).

The National Mental Health Survey conducted in 2015 showed worrying results related to depression and anxiety in adolescents. Suicidal ideation is the most frequent event with 6.6%, followed by the attempt with 2.6% and 1.8% for the suicide plan ( Ministry of Health, 2015).

Between 2009 and 2015 there were 10,325 cases of self-harm, a phenomenon that increased between 2010 and 2016; people aged 15 to 19 comprise the highest rates of attempted suicide ( Ministry of Health, 2017). In the years 2009 to 2017, 2,128,573 schoolchildren and adolescents with diagnoses associated with mental pathologies were attended. With an average of 236,508 people served per year, the trend is towards an increase in cases each year, with a significant decrease in 2016 ( Ministry of Health, 2018).

For Siguenza (2015), the family is considered as the main resource and support group because it is the first contact of an individual with society, which helps adaptation and contact with other social groups. The support that the family provides and allows the person will be essential in their development and balance for both individuals and family ( López, 2009).

The expressions of affection within the family nucleus are considered a family function that favors not only the dynamics but also the family union between parents and children ( Velez and Betancurth, 2016). On the contrary, a dysfunctional family becomes a risk factor for the adolescent, with a greater possibility of risk in their mental health ( Rivera *et al.,* 2018).

Recent studies associated with mental health in adolescents ( Meng *et al.,* 2020; Lee, 2020) bring up the emphasis on these factors, which become particularly relevant due to the processes associated with the situation on the pandemic global, and its behaviors of this adolescent. Especially when the first year of limitations has passed (emphasis in 2020), due to mobility/traffic control, teleworking, online education, and confinements adopted by public administrations, as measures to counteract the spread of such a pandemic.

The more social support adolescents receive there is an improvement in their mental state which favours the maintenance of emotions even in adverse circumstances. This evidence supports the need to propose and implement strategies to promote mental health and increase social support in adolescents ( Gallegos *et al.,* 2020; Galiano *et al.,* 2021).

From the nursing discipline, some different models and theories allow professionals in this area to guide the practice of their professional tasks In this regard, it is pertinent to specifically mention the theory of critical care proposed by Adeline Falk-Rafael in 2005. This theory in turn allows integrating this care from the social determinants of health, facilitating the understanding of various risk factors, and addressing them comprehensively and holistically in the subject of care ( Dickson and Lobo, 2018).

In the method (measurement) subsection, emphasis is placed on three survey/questionnaire instruments to be applied to the target population sample. Likewise, new techniques of data analytics (artificial intelligence) are used to address specific problems associated with the data of such adolescents and how the variables that these represent could be grouped (clustering) in a meaningful way.

In school-aged order, it is hypothesized that the risk of mental health and suicide attempt in adolescents is associated with family functioning. The purpose of this study is to verify the relationship between mental health risk, suicide attempt, and family functionality in a group of adolescents in school-aged 16 to 20 years to cope with relevant interventions based on the N *orth American Nursing Diagnosis Association* (NANDA), Nursing Interventions Classification (NIC), and the Nursing Outcomes Classification (NOC).

## Methods

### Ethical approval

This study was approved by the Research Ethical Committee of the Simón Bolívar University of Barranquilla-Colombia. In compliance with the recommendations of the Committee, the endorsement of the Project from which the postulated article is the product corresponds to CIE-USB-CE-0267-00, legalized on May 24, 2019. The purpose of the study was explained to directors, teachers, parents, and students. Likewise, it was explained all of them about the objective of the research, the ethical, and scientific component for their authorization and participation. The student as participants were told that by accepting their involvement in the study, they would remain anonymous regarding the associated information from the surveys. Once this authorization is obtained, the links of the instruments are delivered to each teacher and selected groups. It is explained that the study constitutes academic information, oriented to the creation of strategies that promote Mental Health Promotion. Permission from the educational institution, informed consent from the parents, and assent from the adolescents were required for this study. Due to the physical limitations of the COVID-19 pandemic, written informed consent was obtained digitally on the project’s webpage.

### Design and sample

The present study is of a correlational, descriptive, and cross-sectional type carried out in a group of adolescents between 16 and 20 years old from 21 district educational institutions in the city of Barranquilla-Colombia. The adolescents were attending the last grade of secondary school and received complementary studies in a technical education institution. The inclusion criterion in this study were that participants had to be Colombian belonging to the age groups of 16 to 20 years and being in secondary school involved in the complementary studies from a program of the Ministry of Education of Colombia ^1^ in the city of Barranquilla-Columbia.

The average number of students per institution was thirty students, for a total of 630.

It is intended to establish the association between risk of mental health, suicide attempt, and family functionality for subsequent strategy design intervention from nursing. The sampling carried out was for convenience or intentional. The selected study subjects were conveniently available to the researchers in one place. As seen in the previous paragraph, the students came from different educational institutions in the city but received complementary technical studies at the same educational institution, which was available to the research group.

The procedure for the selection of the participants was carried out in a technical institution for training for work and human development in a group of 11th-grade of secondary school. This grade was selected because it is the last year of studies that adolescents take before entering university and given the needs of the mental health approach, an immediate nursing intervention plan was required in this group of students. This group of students have been prioritized taking into account the increase in the numbers of mental health problems that affects this age group and the level of studies in which the young people were found, which for the Colombian educational system is the last level before entering to university, which requires preparation for the process of change. The need then arises to detect exposure to risk factors early in order to prevent them with nursing care in Primary Care or refer them for timely management.

The students participating in the study come from different educational institutions in the city. The 21 educational institutions selected that had an agreement with the educational corporation for complementary studies participated. The students of the 21 educational institutions pursue complementary studies in a technical institution ‘CODETEC’ different from the one in which they carry out their regular studies. In this institution they pursue complementary studies according to their interests as assistants in public health, pharmacy, administrative health, beauty, oral health, job security, and customs, among others through an agreement between the Mayor's Office of the city of Barranquilla and CODETEC.

The complementary studies correspond to a program of the Ministry of Education of Colombia that aims to prepare students for job performance in one of the productive sectors and graduate with two degrees: the first accredits them as high school graduates and the second as assistants or technicians of a specific area ^2^. During the process of collecting information, managers, teachers, and students were informed of the objective of the research, ethical, and scientific component for their authorization and participation. Due to the limitations imposed by lockdowns due to the pandemic in the city, in order to achieve access to the adolescents and operators of the questionnaires, a small Google link ( Rojas and Gil, 2020) for presenting the project was developed and, with the support of WhatsApp, it was possible to digitally show the project to both the adolescents participating and the interested community, however the questionnaire was only sent to students as participants.

In this process, participating students have been informed that the questionnaires would be processed anonymously and that the information would only be used for research purposes. Each one of the students was accompanied and informed by their professors regarding completion of the questionnaires, which were delivered through the WhatsApp technological application with prior informed consent. Once this authorization was obtained, the links of the instruments were given to each professor and they in turn deliver it to the students included in the study to be filled out by the participants. The data were collected between June to December 2020.

The total population consisted of 630 students, who have been assigned an identification code, which has been used in each instrument to preserve the identity of the participants which allowed them to be related to each other and to exclude those participants who did not fill out all the three instruments outlined in the measurement section. To estimate the sample size, the statistical equation 1, has been applied.

**Equation 1**: Equation used to determine sample size required for this study.

$$n=\frac{N\;Z^{2}p\;q}{\left(N-1\right)\;E^{2}+Z^{2}p\;q}$$

N = Total population 630 students

Z ^2^ = probabilistic confidence (distribution factor 1.96)

E ^2^ = Error 5% (0.05)

p = 50% (0.5)

q = 50% (0.5)

Sample calculation with the case data:

$$n=\frac{630\times 1.96^{2}\times 0.5\times 0.5}{\left(629\right)\;0.05^{2}+1.96^{2}\times 0.5\times 0.5}$$

$$n=\frac{630\times 3.8416\times 0.25}{\left(629\right)\;0.0025+3.8416\times 0.25}$$

$$n=\frac{605.052}{2.5329}$$

n = 239 (Sample size).

276 students filled out or completed the three instruments, of which 30 samples were excluded because they did not meet the inclusion criteria for filling in all three proposed information collection instruments. Finally, a total of 246 valid samples were obtained; a correlation of the study variables was identified in 37 of them.

The research on the field consisted of five people: one woman and four men. Two of the members are nursing professionals with experience in the disciplinary area, reading the application of models, theories, and nursing process. One of the nursing professionals has community experience in Primary Health Care. Two researchers have Ph.Ds. in Economics and the main doctoral researcher in Computer Science and IT. All of them with numerous publications in high-impact journals. The interdisciplinarity of the team allowed each one to contribute from their investigative and disciplinary experience to the achievement of the main purpose of the investigation of correlating risk variables in mental health, family functionality and suicidal risk to timely detect health problems in adolescents and act from them throw Primary Care for its promotion and prevention.

### Intervention plan

Based on the first results obtained from the application of the instruments proposed for data collecting, in which mental health risk, family dysfunction, and suicide attempt in adolescents were identified, a detailed intervention plan was designed.

For the design of the intervention plan, first, the NNN consult database was consulted, which is an online tool that allows quick consultation of the standardized diagnostic languages developed by NANDA, NOC, NIC and the links between them. For the mental health risk variable, the nursing diagnoses called: tendency to adopt risk behaviors for health and anxiety were selected. Based on the previously selected diagnoses, NOCs for health-promoting behaviors and anxiety self-control were proposed, with their respective evaluation indicators. For the NIC, nursing interventions for health education and anxiety reduction were selected with the activities to be carried out for each case.

In the suicidal risk variable, the chosen NANDA nursing diagnosis was suicidal risk, for the NOC self-control of suicidal impulse and the NIC prevention of suicide. The proposed activities were focused on teaching the patient coping strategies, discuss plans to deal with suicidal ideation and implement actions necessary to reduce immediate distress.

For the family functionality variable, based on NANDA, the nursing diagnosis of dysfunctional family processes, the NOC family functioning, and for the NIC the stimulation of family integrity were prioritized. The main activities proposed were focused on helping the family maintain positive relationships, facilitating communication, facilitating harmony within the family.

117 adolescents participated, who were selected from seven educational institutions where the variables under study were correlated.

All the students who were in grade 11 of the 21 institutions studied were included in this intervention project regardless of whether there was a correlation between the variables so as not to discriminate against the rest of the group in the process and not to point out (mark) adolescents, at-risk and, because it was a strategy of primary health care with a focus on promotion of the health. In which a nursing care plan is established that contains health education interventions and activities to reduce anxiety, suicide prevention, and stimulation of family integrity. Of the 21 educational institutions characterized, 11 institutions were prioritized within which students with connections of the three variables of the study were found: mental health risk, suicide attempt and family functionality.

For the design of the action plan and to propose a relevant educational intervention strategy, the online tool NNN Consult, and NANDA taxonomy were consulted ( NNN Consult, 2017). From this resource, the domains considered most affected were identified, which were supported based on the variables understudy in the following nursing diagnoses: (See Table 1). Similarly, regarding the design of the intervention, the nursing results classification taxonomy (NOC) was applied with its respective indicators and the detailed nursing interventions classification (NIC) with their respective activities. These were selected taking into account the nursing diagnoses identified in the analysis of the results obtained from the data collection instruments: tendency to adopt risk behaviours for health, anxiety, risk of suicide, and dysfunctional family processes.

**Table 1.** Nursing process based on searches with Nanda International, Nursing Outcomes Classification, and Nursing Interventions Classification (NNN Consult).

NANDA			NOC		NIC
Variable	Domain	Nursing diagnosis	Result	Outcome indicators	Interventions and activities
Mental health risk	[1] Health promotion	[00188] Tendency to adopt risky health behaviors	[1602] Health-promoting behavior	[160210] Uses social support to promote health [160205] Uses effective stress reduction techniques [140217] Controls the anxiety response	[5510] **Health education** Activities: 1. Incorporate strategies to enhance self-esteem. 2. Develop written educational materials at an appropriate reading level. 3. Teach strategies that can be used to deal with unhealthy or risky behavior, rather than giving advice to avoid or change the behavior. 4. Help the patient to recognize and express feelings such as anxiety, anger, or sadness.
	[9] Coping/Stress Tolerance	[00146] Anxiety	[1402] Self-control of anxiety	[140219] Identify anxiety triggers	[5820] **Decreased anxiety.** Activities: 1. Help the patient to identify the situations that precipitate anxiety. 2. Instruct the patient on the use of relaxation techniques. 3. Establish recreational activities aimed at reducing tensions.
Suicidal risk	[11] Safety/Protection	[00150] Suicide risk	[1408] Self-control of the suicidal impulse	[140804] Verbalizes suicidal ideas if they exist [140815] Express hop	[6340] **Suicide prevention** Activities: 1. Teach the patient coping strategies (assertiveness training, control of impulsive acts, progressive muscle relaxation), as appropriate. 2. Discuss plans for coping with suicidal ideation in the future (e.g. precipitating factors, who to contact, where to seek help, ways to alleviate impulses for self-harm 3. Implement actions necessary to reduce the individual's immediate distress by negotiate a non-self-harm or safety contract

NANDA			NOC		NIC
Nursing diagnosis	Result	Outcome indicators	Interventions and activities		
Family functionality	[7] Role/Relationships	[00063] Dysfunctional family processes	[2602] Functioning of the family.	[260214] Involves members in conflict resolution. [260211] Creates an environment where members can freely express their feelings	**Stimulation of family integrity.** Activities: 1. Help the family maintain positive relationships. 2. Facilitate open communication between family members. 3. Facilitate harmony within/between the family. 4. Establish a relationship of trust with family members.

Source: self-made by authors.

### Measurement

Considering the proposed objective in terms of establishing the relationship between mental health risk, suicide risk, and family functionality in school adolescents for a subsequent educational proposal, the pertinent literature was first considered.

Second, the main instruments used in primary care in mental health at the national and international level were reviewed based on the following studies “critical review of the instruments for psychiatric evaluation in primary care” ( Tejada *et al.,* 2014), collection instruments and information from the national survey of Mental Health contained in the methodological document national survey of Mental Health ( Ministry of salud, 2015), and the electronic database bank of instruments and methodologies in Mental Health. The Bank of Instruments and Methodologies in Mental Health is a universal and free electronic database developed within CIBERSAM at the beginning of 2008, which aims to collect all the instruments in Spanish related to Mental Health, with the aim of facilitating access to the questionnaires ( Cibersam, 2015).

The search for instruments in the area of mental health was carried out through different filters proposed by the search tool: in the type of population to which it is directed, adolescents and children were selected; in the criteria of therapeutic area, risk factors and projects and diagnosis; in type of alteration, all were selected. The results of this search were organized in an Excel matrix that summarized the following information for each instrument: instrument name, objective, description, author, psychometric properties, and internet link. Those that partially or completely adapted to the needs of the study in terms of early detection of risks in mental health were preselected, excluding those that did not fit the age group of adolescents or psychiatric pathologies themselves.

Initially, 11 instruments used in mental health were preselected: Beck Anxiety Inventory, a useful instrument in early screening for depression or subclinical depression; the Alcohol Consumption Disorders Identification Test (AUDIT), an instrument capable of detecting non-serious problems related to alcohol consumption; the Family Apgar, used to identify families with possible dysfunctions; The Altman Self-Rating Mania Scale (ASM) is a brief scale developed to measure the presence and severity of manic symptoms; Anxiety Screening Questionnaire (ASQ-15), useful for detecting generalized anxiety disorders; Forages Adult Self-Report 18-59 (ASR 18-59), the ASR for adults aged 18-59 belongs to the assessment system to assess psychopathology; Scale of Interpersonal Difficulties for Adolescents, the EDIA scale evaluates the perception of adolescents about the level of difficulty of the situations; Generalized Anxiety Disorder (GAD-7), this scale assesses the severity of anxiety globally in patients who meet criteria for anxiety or depression; Suicide Risk Assessment Scale (ERS) evaluation of the risk of suicide in adolescents; Adult Psychiatric Symptom Self-Report Questionnaire (SRQ), this instrument provides the ability to determine the user's health status and assess the presence of a condition that may be related to mental health; Symptom questionnaire for children – RQC (Reporting Questionnaire for Children) useful to identify children and adolescents between 5 and 15 years old, who present symptoms compatible with possible mental disorders; GHQ-12 The GHQ-12 is an effective screening measure for assessing psychological well-being and detecting non-psychotic psychiatric problems in people; ASSIST V3.0 (Alcohol, Tobacco and Substance Use Screening Test). Those instruments that evaluated psychiatric pathologies, not applicable to the age group under study, not exclusive to Primary Health Care or those that only allowed the identification of a specific risk factor were excluded. For the inclusion criteria in the selected instruments, their applicability in Primary Health Care and the identification of risks in mental health and family functionality were taken into account.

After reading the description of each of them and the purpose of their application, three instruments were selected: the Family Apgar, the Suicide Risk Assessment Scale (ERS), and the Self-Report Questionnaire of Psychiatric Symptoms in Adults (SRQ), which were consulted as valid by three academic experts in the area ( Rojas *et al.,* 2022). The variables evaluated with the three selected instruments are listed below. The experts were identified for their expertise in the area of mental health. The professionals were two nurses and a psychologist who not only participated in the selection of the data collection instruments, but also in the planning of the nursing care plan, suggesting interventions and activities to address the diagnoses identified in adolescents.

*Mental health risk*

For the analysis of this variable, the self-reporting questionnaire (SRQ) of psychiatric symptoms ( Ministry of salud, 2020), was used. No changes were made to the instrument for the present study. The instruments were translated to English in the extended data after data collection ( Rojas *et al.,* 2022). This instrument measures five specific areas: depression, anxiety, alcoholism, psychosis, and epilepsy. It is applied to adolescents from 16 years of age onwards and consists of 30 questions with answer options of YES and NO. It consists of two parts: an initial one with identification data about the respondent, a block of 20 questions on non-psychotic psychiatric symptoms (anxious/depressive); a second segment of 10 questions that refer to psychiatric symptoms of a psychotic type, convulsive or alcohol consumption.

*Family functionality*

To identify the type of family function, the family APGAR instrument was used ( Suarez Cuba and Espinoza, 2014). To identify the type of family function, the family APGAR instrument was used ( Suarez Cuba and Espinoza, 2014). This instrument was designed in 1978 by Dr. Gabriel Smilkstein, who, based on his experience as a family doctor, proposed the application of this test as a tool for professionals in the area of primary health care, in their analysis approach. of the family role. This test is based on the premise that family members perceive the functioning of the family and can express the degree of satisfaction with the fulfillment of its basic parameters.

This test has called ‘family APGAR’ because it is an easy word for health professionals to remember, given the similarity with the nearly universally used test in newborn screening proposed by Dr. Virginia Apgar. This instrument evaluates five basic functions of the family: adaptation, participation, gain, affection, and resources. Each of the responses has a score that ranges between 0 and 4 points. When adding the five parameters, the score fluctuates between 0 and 20, which indicates good family function, moderate family dysfunction, mild family dysfunction, and severe family dysfunction.

*Suicide risk*

Suicidal risk assessment was carried out using the instrument for suicide risk assessment in adolescents. It is a Likert-type scale designed and validated in Colombia by psychologists Marly Bahamón and Yolima Alarcón in their research work ( Bahamón and Alarcón, 2018). It evaluates these factors: 1) depression and hopelessness, 2) ideation, planning, and self-harm, 3) isolation/social support, and 4) lack of family support. The variables high, medium, and low were defined in the global suicide risk score according to the authors' instructions. described in the document entitled design and validation of a scale to assess the risk of suicide (ERS) in Colombian adolescents provided by the authors at the time of authorizing the use of the ERS scale for research purposes.

As a research pilot test, the questionnaires proposed for this research were previously applied to a small sample corresponding to 29 adolescents enrolled in school, in order to detect biases in the processing and analysis of information. The adolescents selected for the pilot test were school students from the same participating educational institutions, they were not excluded from the main study. The selection of the sample was random with a raffle to select the educational institution and the participants for the pilot and for the convenience of the different educational institutions, the links of the questionnaires were distributed via Whatsapp to the participants who in turn filled them out.

The results obtained made it possible to identify that there was no way to relate the results of the instruments: family functionality/mental health risk/suicide risk. Based on this analysis, the identification document number is included in the instruments but in processing it is replaced by a sequential numerical code to preserve its identity. In this way, not only the previous aspect is corrected, but also the information of adolescents who did not complete the three proposed instruments: SRQ, family APGAR, and ERS scale is also excluded.

### Data analytics strategy

To establish the association of the variables studied, the results obtained were analysed using machine learning techniques (clustering), which are tools that help to understand the different subgroups that exist within the data set. These techniques aim to group elements that are close enough to each other and far enough from other elements ( Rokach and Maimon, 2005).

Therefore, the Gower distance technique was used which is a measure of distance that can be calculated for two individuals whose attributes are mixed. The Gower distance is calculated as the average of the differences between individuals. Each Gower distance lies between [0,1]. The partial dissimilarity d (i, j) (f) depends on the type of variable we are measuring. In the case of numerical variables, partial dissimilarity is the relationship between the absolute differences between the observations and the maximum observed range of all individuals. In the case of categorical variables, the partial dissimilarity is 1 if the observations are different and 0 otherwise ( Asensio *et al.,* 2022). The formula applied is the following in the equation 2

$$d\left(i,j\right)=\frac{1}{p}\sum_{i=1}^{p}d_{\text{if}}^{\left(f\right)}$$

**Equation 2:** Gower distance equation used in this study.

The clustering algorithm selected should fit well with the Gower distance. For this, the *k*-medoids algorithm was selected, which is a classic partitioning method like the well-known *k*-means method but, instead of iterating over the centroids, it iterates over the medoids that is, it tries to find the most representative object of each group ( Rokach and Maimon, 2005).

The algorithm groups the objects into a total of k groups where k must be given *a priori.* The selection of the optimal number of clusters (k) should be made considering the statistical information obtained in the data, although if there is some reasoned justification *a priori*, the number of clusters may vary for different reasons. For the optimal number of clusters to divide our data into, we use the width of the silhouette. The width of the silhouette is one of the most used options to measure the similarity between each point of a group and compares this similarity with the closest point of the neighbouring group. This metric is between [-1, 1] where higher values mean greater similarities ( Asensio *et al.,* 2022).

**Figure 1.**
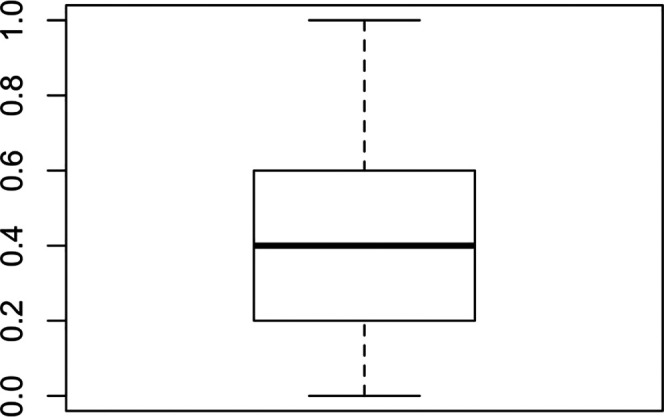
Gower model optimal clusters. Source: self-made by authors.

Figure 1 shows the result of the measurement for values of k between 2 and 10 where it can be observed that segmenting the students into eight groups of five or six maximizes the similarity within the clusters and the dissimilarity between the clusters. The sample has been divided into eight groups following the results found in the silhouette analysis.

**Figure 2.**
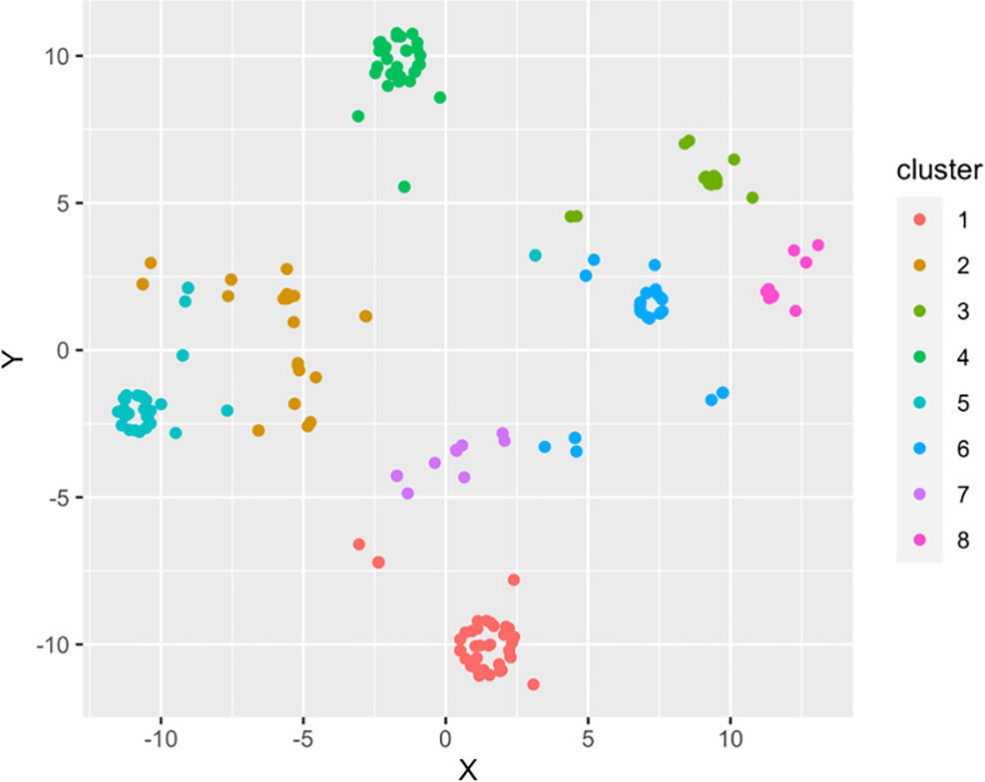
Viewing Gower clusters. Source: self-made by authors.

This result can be seen in Figure 2, a visualization with the observations classified in six clusters.

Now comparing the characteristics, it can be seen where the differences are concerning surveyed students and with data that can be used for proper segmentation, to apply intervention strategies, The group represented in Table 2 are those that correspond to these groupings resulting from applying the algorithm, which will become a focus group (of interest or reference) for the research.

**Table 2.** Focal group selected by Gower's algorithm.

N°	Suicide risk	family functionality	Significant distress (Yes/No)	Psychotic symptoms (Yes/No)	Alcohol consumption (Yes/No)	Cluster
*1*	Tall	Dysfunction-severe	Yes	Yes	Do-not	6
*2*	Tall	Dysfunction-severe	Yes	Yes	Do-not	6
*3*	Tall	Dysfunction-severe	Yes	Yes	Do-not	6
*4*	Half	Dysfunction-severe	Yes	Yes	Do-not	6
*5*	Half	Dysfunction-severe	Yes	Yes	Do-not	6
*6*	Half	Dysfunction-severe	Yes	Yes	Do-not	6
*7*	Half	Dysfunction-severe	Yes	Yes	Do-not	6
*8*	Half	Dysfunction-severe	Yes	Yes	Do-not	6
*9*	Half	Dysfunction-severe	Yes	Yes	Do-not	6
*10*	Half	Dysfunction-severe	Yes	Yes	Do-not	6
*11*	Half	Dysfunction-severe	Yes	Yes	Do-not	6
*12*	Half	Dysfunction-severe	No	Yes	Do-not	6
*13*	Half	Dysfunction-severe	No	Yes	Do-not	6
*14*	Half	Dysfunction-severe	Yes	Yes	Do-not	6
*15*	Half	Dysfunction-severe	Yes	Yes	Do-not	6
*16*	Half	Dysfunction-severe	Yes	Yes	Do-not	6
*17*	Half	Dysfunction-severe	Yes	Yes	Do-not	6
*18*	Half	Dysfunction-severe	Yes	Yes	Do-not	6
*19*	Half	Good-function	Yes	Yes	Do-not	6
*20*	Half	Dysfunction-severe	Yes	Yes	Do-not	6
*21*	Half	Good-function	Yes	Yes	Do-not	6
*22*	Half	Dysfunction-severe	Do-not	Yes	Do-not	6
*23*	Half	Dysfunction-severe	Yes	Yes	Do-not	6
*24*	Half	Dysfunction-severe	Do-not	Yes	Yes	6
*25*	Half	Dysfunction-severe	Do-not	Yes	Do-not	6
*26*	Half	Dysfunction-severe	Yes	Yes	Do-not	6
*27*	Low	Good-function	Yes	Yes	Do-not	6
*28*	Low	Dysfunction-severe	Yes	Yes	Do-not	*6*
*29*	Low	Dysfunction-severe	Yes	Yes	Do-not	6
*30*	Low	Dysfunction-severe	Do-not	Yes	Do-not	6
*31*	Low	Dysfunction-severe	Yes	Yes	Do-not	6
*32*	Low	Dysfunction-severe	Yes	Yes	Do-not	6
*33*	Low	Good-function	Yes	Yes	Do-not	6
*34*	Low	Dysfunction-severe	Yes	Yes	Do-not	6
*35*	Low	Dysfunction-severe	Yes	Yes	Do-not	6
*36*	Low	Good-function	Yes	Yes	Do-not	6
*37*	Low	Good-function	Yes	Yes	Do-not	6

Source: self-made.

To improve the reliability and credibility of the information, the verification of the participants has been established through interaction in remote meetings during the class sessions in order to also contrast the results obtained with what the students said and thought about the subject under investigation. Following the methodological rigor proposed by Guba and Lincoln (1981) in terms of auditability, the skills of the study researchers have been used for the respective follow-up. In this way, the data was examined by the different researchers, reaching complementary contributions and conclusions around the research topic.

## Results

A total of 246 adolescents were included in this study after completed all three tools in full. In relation to the socio-demographic variables studied of sex and age in the 246 adolescents studied, there is a higher proportion of women (65%) compared to men (35%). The participants were aged 16 years (33%), 17 years (42%), 19 years (6%), and 20 years (5%) respectively ( Rojas *et al.,* 2022).

Regarding the non-psychotic psychiatric symptoms of the SRQ instrument, it was found that the most frequently reported symptoms were disinterest (44%), nervousness (37%), trouble thinking clearly (37%), indecision (37%), headache (33%), and fatigue (25%). The most frequently reported psychotic psychiatric symptoms were the belief that your feelings are more important than what others think (76%) and the feeling that someone has tried to hurt you (40%). These results indicate significant psychological pain (28%), psychotic-type symptoms (85%), and problematic alcohol consumption (9%).

Concerning the family functionality obtained from the application of the family APGAR. The good family function was 34% while the remaining 66% presented some type of family dysfunction: mild family dysfunction (31%), moderate family dysfunction (21%), and severe family dysfunction (15%).

Regarding the global suicide risk score in the surveyed adolescents, it was found that 74% presented a low risk for this variable, while 24% presented a medium risk, and 2% showed a high risk. When analyzing the relationship between the variables of the present study, it was possible to show that there is a significant correlation in 15% (37) of those surveyed (See Table 3).

**Table 3.** Correlation of variables.

Suicide risk			Family function			Mental health risk					
					Significant distress		Psychotic symptoms		Problem alcohol use		
Tall	Half	Under	Good	Severe	Yes	No	Yes	No	Yes	No	
3	0	0	0	3	3		3	0	0	3	
0	23	0	2	21	17	6	23	0		1	22
0	0	11	4	7	10	1	11	0		0	11
3	23	11	6	31	30	7	37	0		1	36

Source: self-made.

In three adolescents, a high suicide risk, severe family dysfunction, and mental health risk were found; specifically, with symptoms indicating distress and psychotic symptoms. Similarly, a marked correlation was identified in twenty-three adolescents with medium suicide risk, and in eleven of them a low suicide risk and correlation of the same variables ( Rojas *et al.,* 2022).

## Discussion

The findings are related to preliminary studies that show the prevalence of the female gender to present symptoms related to anxiety crisis, mood alterations, deep sadness among other symptoms compared to the male gender ( Riecher-Rossler, 2017; Odéhn and Goulding, 2018; Chaskel *et al.,* 2015). This may be a trend in this population group (female) due to family overload and psycho-social stressors ( Sol-Pastorino *et al.,* 2017).

Regarding the clinical characteristics related to non-psychotic psychiatric symptoms expressed by the participants such as headache, nervousness, disinterest, confusion, fatigue, and psychotic type, such as feeling that they have tried to hurt and primarily significant psychological anguish, the capacity for self-recognition is striking adolescents themselves, which reflects the felt need for mental health. In this regard ( Gómez-Rest EPO *et al.,* 2021) expresses that the perception of inadequate mental health would gain importance because it allows the professional involved room for planning and a timely approach, especially in this stage of life, in which they are going through various processes and changes of a biological, psychological, and social nature.

In González *et al.* (2017) and Guo *et al.* (2018) studies, they highlight both the importance of family support as well as the negative influence of family stressors such as intra-family violence, inappropriate parental roles, and the breakdown of family dialogue as risk factors in adolescent mental health.

In the case of suicidal behaviour, a major trigger is a depression. Approximately 60% of people with depression have thought of suicide as a way out of their experiences and 30% attempted suicide ( Rey *et al.,* 2015). This behaviour is potentiated if there are family stressors, aggressions, loneliness, or suicidal behaviours in the family nucleus, among other related factors.

Wang *et al.* (2020) state that adolescents with high degrees of family dysfunction are more exposed to suffering psychological conditions that can affect their mental health and trigger a high suicide risk. Some scientific literature confirms the relationship of the suicide attempt with factors such as anxiety, traumatic events, and the social environment, which has a significant impact on the public health of a region ( Arenas *et al.,* 2016).

The authors ( Radovic *et al.,* 2015; Rey *et al.,* 2015) suggest motivational therapies between parents and children as the main recommendations for family functioning which provides adolescents with security, resources, and strengths to face challenges, demands, and threats to their mental health. The strengthening of emotional skills is considered a key element that undoubtedly improves not only their self-esteem but also their perspectives towards their life project ( Bonet *et al.,* 2020).

The strengthening of the primary level of health care is required, as well as community empowerment and interdisciplinary work. In the same way, consider the training of human talent in health. The use of technologies also becomes a key factor specific to this age group with ‘e-mental health’ or ‘telepsychiatry’ ( Rojas-Bernal *et al.,* 2018).

Due to the fact that these types of technological tools cause good receptivity in these age groups, they offer multiple benefits not only in education and health promotion, but also in prevention, diagnosis, treatment and recovery. They become a challenge for health professionals, and especially for nurses who are in charge of direct care, leadership and management in health promotion and maintenance programs.

### Research limitations

The ability to determine whether the correlation between suicide risk, mental health risk, and family dysfunction has been influenced by current circumstances, derived from confinement due to the pandemic and the underlying climate. Although the corresponding methodological process and explanations were carried out in the completion of the information collection instruments, these were completed online, which may cause bias in the results obtained.

## Conclusion

The results obtained suggest an interdisciplinary approach given the correlation between suicide risk, mental health risk, and severe family dysfunction in a significant group of scholarhood adolescents. It is important to emphasize the need for urgent attention to them, firstly because of the repercussions that these have on the age group studied and secondly, because of the degree of chronicity they represent in adulthood, presenting problems and conditions not only of a biological nature but also of a social and family one.

In the same way, it is evidenced that the integration of the parental role and family dynamics play a fundamental role in the actors involved, not only in prevention but maintenance, recovery, and rehabilitation in adolescents. This process should not be isolated but must include the school, the family, and the adolescent.

## Data availability

### Underlying data

Zenodo. Mental health, suicide attempt, and family function for adolescents' primary health care during the COVID-19 pandemic. https://doi.org/10.5281/zenodo.6466538 ( Rojas *et al.,* 2022).

The project contains the following underlying data:

• Adult Psychiatric Symptoms Self-Report Questionnaire (SRQ). (Anonymised results from the SRQ questionnaire with English and Spanish versions)
• Suicide risk data. (Anonymised results from the suicide risk questionnaire with English and Spanish versions)
• Family Apgar Instrument. (Anonymised results from the family Apgar questionnaire with English and Spanish versions)
• Consolidated instruments results. (This file contains the consolidation of the results of the three instruments applied to the population under study with English and Spanish versions)
• Consolidated codified instruments. (This file contains the consolidation and coding of data from the three instruments used in the present study

Data are available under the terms of the Creative Commons Attribution 4.0 International license (CC-BY 4.0).

### Extended data

Zenodo. Mental health, suicide attempt, and family function for adolescents' primary health care during the COVID-19 pandemic. https://doi.org/10.5281/zenodo.6466538 ( Rojas *et al.,* 2022).

• Figure 1. Gower model optimal clusters. (Figure 1 shows the measurement result for values. Similarity within clusters and dissimilarity between clusters are maximized.)
• Figure 2 Viewing Gower Clusters. (Figure 2 provides relevant information organized into six 6 clusters. It can be seen where the differences are in terms of the students surveyed and with data that can serve for an adequate segmentation, to apply intervention strategies)
• Table 1. Nursing process based on searches NNN Consult. (Table 1 shows the nursing care plan with the diagnoses, interventions and activities to be carried out, as well as the objective to be achieved according to the needs identified in the participants)
• Table 2. Focal group selected by Gower's algorithm. (Table 2 shows the results of the focus group selected by the Gower algorithm)
• Table 3. Correlation of variables. (Table 3 shows the correlation of variables of the three instruments applied to the study participants)
• Self report questionnaire. (Blank copy of the self-reporting questionnaire with English and Spanish versions).
• ERS Suicidal Risk scale. (Blank copy of the ERS suicidal risk scale with English and Spanish versions)
• Family Apgar instrument. (Blank copy of the Family Apgar instrument with English and Spanish versions).
• Annex web link of project information. (Link to send information on project to participants, parents, and institutions)

Data are available under the terms of the Creative Commons Attribution 4.0 International license (CC-BY 4.0).

## Reporting guidelines

SRQR checklist for ‘Mental health, suicide attempt, and family function for adolescents' primary health care during the COVID-19 pandemic [Data set]. Zenodo. https://doi.org/10.5281/zenodo.6466538 ( Rojas *et al.,* 2022).

Data are available under the terms of the Creative Commons Attribution 4.0 International license (CC-BY 4.0).

## Footnotes

^1^
More information available at https://www.mineducacion.gov.co/1780/w3-propertyvalue-56741.html?_noredirect=1 and http://200.24.48.52/educa/index.php/doble-titulacion .

^2^

http://200.24.48.52/educa/index.php/doble-titulacion
