# Supplementary material for: Mental health, suicide attempt, and family function for adolescents’ primary health care during the COVID-19 pandemic
Source: F1000Res. 2022 Sep 23;11:529. Originally published 2022 May 16. [Version 2] doi: 10.12688/f1000research.109603.2 (PMC9751494; doi:10.12688/f1000research.109603.2)
Supplement: Peer review history

# Peer review: Reviewer response for version 2

**DOI:** 10.5256/f1000research.138866.r151264

**Published:** 2022-12-14

**Authors:** Sebastián Gabini

**Affiliations:** 1 Universidad Abierta Interamericana, Centro de Altos Estudios en Ciencias Sociales (CAECS), Rosario, Argentina

Some of the suggested changes to the article have been made. Which improved its quality. It is believed that the article can be approved. However, the need to review the analysis of correlations is highlighted. Now, it responds more to a contingency table (Table 2) than to a correlational analysis between the variables. For this, one of the appropriate statistics must be used, such as Pearson's r or Spearman's rho, depending on the normality of the data.

Is the work clearly and accurately presented and does it cite the current literature?

Yes

If applicable, is the statistical analysis and its interpretation appropriate?

Partly

Are all the source data underlying the results available to ensure full reproducibility?

Partly

Is the study design appropriate and is the work technically sound?

No

Are the conclusions drawn adequately supported by the results?

Partly

Are sufficient details of methods and analysis provided to allow replication by others?

Yes

Reviewer Expertise:

Psychology, mental health, mental health in workplace, psychometric assessment, quantitative analysis.

I confirm that I have read this submission and believe that I have an appropriate level of expertise to confirm that it is of an acceptable scientific standard.

# Peer review: Reviewer response for version 2

**DOI:** 10.5256/f1000research.138866.r151265

**Published:** 2022-09-30

**Authors:** Valentina Lucia La Rosa

**Affiliations:** 1 Department of Educational Sciences, University of Catania, Catania, Italy

No further changes are needed

Is the work clearly and accurately presented and does it cite the current literature?

Partly

If applicable, is the statistical analysis and its interpretation appropriate?

Yes

Are all the source data underlying the results available to ensure full reproducibility?

Yes

Is the study design appropriate and is the work technically sound?

Yes

Are the conclusions drawn adequately supported by the results?

Yes

Are sufficient details of methods and analysis provided to allow replication by others?

Yes

Reviewer Expertise:

Clinical psychology; Mental health; Developmental psychology.

I confirm that I have read this submission and believe that I have an appropriate level of expertise to confirm that it is of an acceptable scientific standard.

# Peer review: Reviewer response for version 1

**DOI:** 10.5256/f1000research.121123.r147902

**Published:** 2022-09-06

**Authors:** Sebastián Gabini

**Affiliations:** 1 Universidad Abierta Interamericana, Centro de Altos Estudios en Ciencias Sociales (CAECS), Rosario, Argentina

The research problem is clear and current. The rationale is well developed and contains relevant citations. The description and selection of the sample, the selection of the measurement instruments and the ethical considerations are consistent. Although in the SRQ instrument the criterion to determine possible psychosis is too narrow (and this is reflected in the results).

 However, methodological design contains some aspects to review. It is maintained that it is a correlational study but no specific statistics are applied to assess it (eg.: Pearson or Spearman correlation). In this case, the type of instrument (which gives quantitative raw scores as a result) would suggest the application of a statistic of this style, rather than a cluster analysis that tends to form groups. Table 3 (referred to as correlation between variables) contains the absolute frequencies for each group. If you choose to work with contingency tables to see the relationship between the variables, you should use statistics such as the chi-square. Note, for example, that about 65% of those who scored low on suicidal risk were from a dysfunctional family.

 Finally, the proposed intervention plan could be part of the study's suggestions but not within the methodology chapter.

 Additionally, it is suggested to review the use of English.

Is the work clearly and accurately presented and does it cite the current literature?

Yes

If applicable, is the statistical analysis and its interpretation appropriate?

Partly

Are all the source data underlying the results available to ensure full reproducibility?

Partly

Is the study design appropriate and is the work technically sound?

No

Are the conclusions drawn adequately supported by the results?

Partly

Are sufficient details of methods and analysis provided to allow replication by others?

Yes

Reviewer Expertise:

Psychology, mental health, mental health in workplace, psychometric assessment, quantitative analysis.

I confirm that I have read this submission and believe that I have an appropriate level of expertise to confirm that it is of an acceptable scientific standard, however I have significant reservations, as outlined above.

# Peer review: Reviewer response for version 1

**DOI:** 10.5256/f1000research.121123.r137986

**Published:** 2022-07-11

**Authors:** Valentina Lucia La Rosa

**Affiliations:** 1 Department of Educational Sciences, University of Catania, Catania, Italy

I read with great interest the Manuscript titled “Mental health, suicide attempt, and family function for adolescents’ primary health care during the COVID-19 pandemic”, which falls within the aim of F1000 Research.    

 In my honest opinion, the topic is interesting enough to attract the readers’ attention. The Authors aimed to identify associations between mental health risk, suicide attempts, and family function in a sample of 246 adolescents. The results underlined that the risk of mental health deterioration and suicide during the pandemic was related to family functionality. The study methodology is adequate, and the conclusions are consistent with the reported data. The manuscript can be improved expanding the references and citing some recently published articles on this topic.

 Authors should consider the following recommendations:

- I recommend a linguistic revision of the Manuscript to improve its readability.
- I recommend better describing the scales used in the study and further specifying their psychometric properties.
- Inclusion/exclusion criteria should be better clarified. More specifically, it is unclear whether the authors excluded adolescents with psychiatric or psychological comorbidities from the sample. I recommend specifying this point more clearly as it could constitute a bias for the study results.
- I suggest citing other studies that have investigated the psychological well-being of adolescents during the pandemic in other countries to compare the authors' findings with those of these studies. Some suggestions are the following: doi:10.3389/fpsyg.2020.559951; doi: 10.1016/j.psychres.2020.113264

Is the work clearly and accurately presented and does it cite the current literature?

Partly

If applicable, is the statistical analysis and its interpretation appropriate?

Yes

Are all the source data underlying the results available to ensure full reproducibility?

Yes

Is the study design appropriate and is the work technically sound?

Yes

Are the conclusions drawn adequately supported by the results?

Yes

Are sufficient details of methods and analysis provided to allow replication by others?

Yes

Reviewer Expertise:

Clinical psychology; Mental health; Developmental psychology.

I confirm that I have read this submission and believe that I have an appropriate level of expertise to confirm that it is of an acceptable scientific standard, however I have significant reservations, as outlined above.

## References

[1] : Adolescents in Quarantine During COVID-19 Pandemic in Italy: Perceived Health Risk, Beliefs, Psychological Experiences and Expectations for the Future. *Front Psychol* .2020;11: 10.3389/fpsyg.2020.559951 559951 10.3389/fpsyg.2020.559951 33071884PMC7538632

[2] : Adolescent psychiatric disorders during the COVID-19 pandemic and lockdown. *Psychiatry Res* .291: 10.1016/j.psychres.2020.113264 113264 10.1016/j.psychres.2020.113264 32622172PMC7323662
